# Conjugated polymers containing diketopyrrolopyrrole units in the main chain

**DOI:** 10.3762/bjoc.6.92

**Published:** 2010-08-31

**Authors:** Bernd Tieke, A Raman Rabindranath, Kai Zhang, Yu Zhu

**Affiliations:** 1 Department of Chemistry, University of Cologne, D-50939 Cologne, Germany

**Keywords:** conjugated polymer, diketopyrrolopyrrole, electroluminescence, photoluminescence, solar cell

## Abstract

Research activities in the field of diketopyrrolopyrrole (DPP)-based polymers are reviewed. Synthetic pathways to monomers and polymers, and the characteristic properties of the polymers are described. Potential applications in the field of organic electronic materials such as light emitting diodes, organic solar cells and organic field effect transistors are discussed.

## Introduction

A useful strategy in the design of new polymers for electronic applications is to incorporate chromophores which are highly absorbing and emitting in the visible and near infrared region into π-conjugated polymers chains. Potentially useful chromophores for electronic applications can be found among the various organic colourants, especially in the field of so-called “high-performance pigments” developed in the last two or three decades [1]. Among these pigments are 2,5-diketopyrrolo[3,4-c]pyrrole (DPP) derivatives, which were commercialized in the 1980s [2–3]. DPPs are the subject of many patents, despite the fact that for a considerable time there were only a few publications that dealt with these compounds.

In recent years, a growing number of polymer chemists and physicists have become interested in DPPs since it was shown that DPP-containing polymers exhibit light-emitting and photovoltaic properties. The purpose of the present article is to review recent activities regarding the deeply coloured, and in many cases, fluorescing polymers. Synthetic pathways, characteristic properties, and possible applications are described.

## Review

### DPP-based monomers

**Figure 1:**
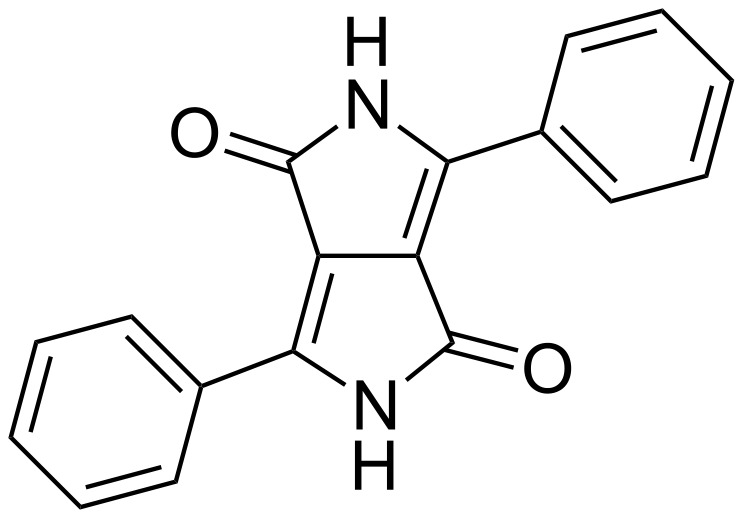
Structure of 3,6-diphenyl-substituted 2,5-diketopyrrolo[3,4-c]pyrrole (DPP).

After the 3,6-diphenyl-substituted DPP (diphenylDPP) (Figure 1) was first synthesized in low yield by Farnum et al. in 1974 [4], Iqbal, Cassar, and Rochat reported an elegant synthetic pathway for DPP derivatives in 1983 [5–6]. It was discovered that DPP derivatives could be prepared in a single reaction step in high yield by the reaction of benzonitrile (or other aromatic nitriles) with succinic acid diesters. Numerous DPP derivatives have since been synthesized, their colours ranging from orange yellow via red to purple. Many DPP derivatives exhibit a high photostability in the solid state, weather fastness, deep colour, luminescence with large Stokes-shifts, and a brilliant red colour enabling technical applications in colouring of fibers, plastics and surface coatings such as prints or inks.

The electron-withdrawing effect of the lactam units causes the chromophore to have a high electron affinity. Strong hydrogen bonding between the lactam units favors the chromophores forming physically cross-linked chain structures in the solid state, which is the origin for the poor solubility [7–8]. Short distances between the chromophore planes (0.336 nm) and phenyl ring planes (0.354 nm) enable π-π-interactions via molecular orbital overlapping and excition coupling effects [7–9], whilst electronic interactions and strong intermolecular forces lead to a high thermal stability of up to 500 °C.

**Scheme 1:**
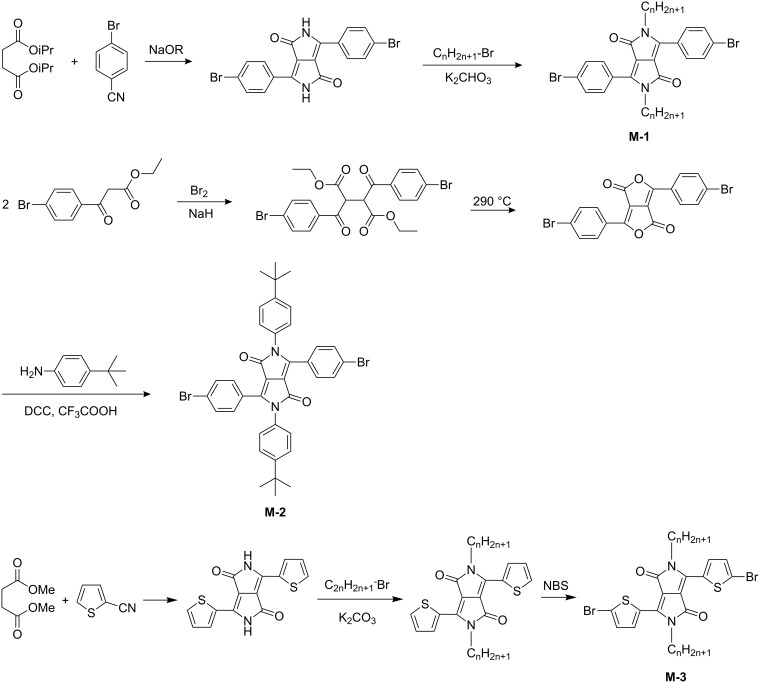
Synthesis of DPP monomers.

For chemical incorporation into conjugated polymers, the solubility of the DPP compound needs to be increased, and the chromophore requires to be functionalized with polymerizable groups. The solubility can be increased by *N*-alkylation [10], arylation [11] or acylation [12] of the lactam units thus preventing hydrogen bond formation between the chromophores. Polymerizable groups can be attached to the aryl units in the 3- and 6-positions of the central DPP chromophore [13], or to the lactam substituent groups [14–15]. Suitable polymerizable groups are halogen atoms (especially bromine and iodine), hydroxyl, trifluoromethylsulfonate, or aldehyde groups. Synthetic strategies recently described are outlined in Scheme 1. For the preparation of brominated diphenyl-DPPs it is necessary to start from bromobenzonitrile and a succinic acid ester and to prepare first the dibromophenyl-DPP pigment, which is subsequently N-alkylated to yield the soluble dibromo-dialkyl-DPP monomer **M-1**. While the N-alkylation of DPP proceeds directly in good yield, the introduction of aryl units in most cases requires a specific synthetic pathway. First, the corresponding diketofurofuran (lactone) compound has to be synthesized [11]. The lactone is subsequently converted into the N-aryl-lactam **M-2** by reaction with an arylamine. The bromination of aryl units is important for the subsequent palladium-catalyzed coupling reaction. If the aryl unit is thiophene, direct bromination with N-bromosuccinimide is possible to yield monomer **M-3** [16].

For the preparation of conjugated DPP-based polymers, palladium-catalyzed polycondensation reactions such as Suzuki [17], Stille [18] and Heck [19] coupling are especially useful. Other suitable reactions are Ni-mediated Yamamoto coupling [20], Sonogashira coupling [21], or electrochemical polymerization [22]. In the following, a brief review of recently prepared DPP based polymers is presented.

### DPP-based polymers

The first DPP-based polymer was described by Yu et al. in 1993 [13]. Conjugated block copolymers containing phenylene, thienylene and N-alkyl substituted diphenyl DPP units in the main chain were synthesized by Stille coupling. Photorefractive polymers were prepared containing a conjugated main chain and nonlinear optically active (nlo) chromophores in the side chain. DPP was incorporated in the polymers as a sensitizer for charge carrier generation. Some years later, Eldin and coworkers described DPP-containing polymers obtained by radical polymerization of bis-acryloyl-substituted DPP derivatives [14–15]. Polymer networks containing non-conjugated, copolymerized DPP units were prepared, whilst linear DPP-containing polyesters and polyurethanes were first described by Lange and Tieke in 1999 [23]. The polymers were soluble and could be cast into orange films that exhibited a strong fluorescence with maxima at 520 nm and a large Stokes-shift of 50 nm. However, due to the aliphatic structure of the main chain, the thermal stability was rather poor. Photoluminescent polyelectrolyte-surfactant complexes were obtained from an amphiphilic, unsymmetrically substituted DPP-derivative upon complex formation with polyallylamine hydrochloride or polyethyleneimine [24]. The complexes exhibit a mesomorphous structure with the glass transition temperatures dependent on the structure of the polyelectrolyte.

The first synthesis of conjugated DPP-polymers and copolymers via Pd-catalyzed Suzuki coupling was reported by Tieke and Beyerlein in 2000 [25]. The polymers contained *N*-hexyl-substituted diphenylDPP units and hexyl-substituted 1,4-phenylene units in the main chain and molecular weights of up to 21 kDa were determined. Compared with the monomer, the optical absorption of the polymer in solution was bathochromically shifted by 12 nm with the maximum at 488 nm. The polymer also showed a bright red fluorescence with the maximum at 544 nm. In addition to the alternating copolymer, copolymers with lower DPP content were also prepared. All copolymers showed the DPP absorption at 488 nm, the ε-value being a linear function of the DPP content. Upon UV irradiation the copolymers gradually decomposed. The rate of photodecomposition was found to increase with decreasing DPP phenylene comonomer ratio. Two different photoprocesses were recognized: a slow process originating from the absorption of visible light by the DPP chromophore, and a rapid one arising from additional absorption of UV-light by the phenylene comonomer unit followed by energy transfer to the DPP chromophore. The actual mechanism of photodecomposition remains unclear. Comparative studies indicated that conjugated DPP-containing polymers are considerably more stable than the DPP monomers or non-conjugated DPP-polymers.

**Figure 2:**
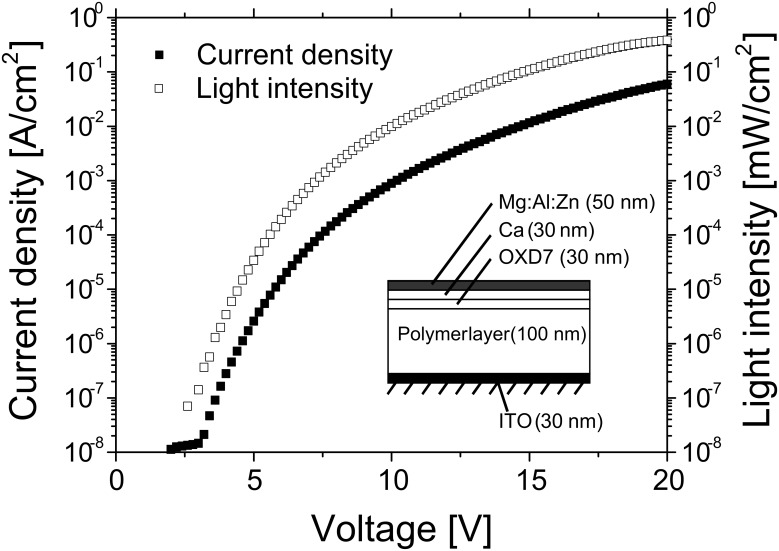
Plot of current density and light intensity versus voltage of polymer light-emitting diode containing **P-5**: ITO/**P-5**/OXD7/Ca/Mg:Al:Zn (from [28]).

Dehaen et al. used a stepwise sequence of Suzuki couplings to prepare rod-like DPP-phenylene oligomers with well-defined lengths [26]. The resulting oligomers contained three, five and seven DPP units, respectively. Unfortunately, the effect of the chain length on absorption and emission behaviour was not reported. A study on thermomesogenic polysiloxanes containing DPP units in the main chain was published in 2002 [27]: Investigations on the thermotropic phase behaviour using polarizing microscopy revealed nematic and smectic enantiotropic phases. In the same year, the first study on electroluminescent (EL) properties of a DPP-containing conjugated polymer was reported by Beyerlein et al. [28] who studied a DPP-dialkoxyphenylene copolymer in a multilayer device of ITO/DPP-polymer/OXD7/Ca/-Mg:Al:Zn and observed a red emission with maximum at about 640 nm. A relevant plot of current density and light intensity vs. voltage is reproduced in Figure 2. DeSchryver et al. synthesized dendrimer macromolecules with a DPP core [29]. Embedded in a spin-coated polystyrene film, single dendrimer molecules could be imaged via a confocal microscope by utilizing the strong fluorescence of the DPP core. It could be shown that the orientation of the absorption transition dipole of single dendrimer molecules in the film changed in a time window of seconds.

### Recent work on diphenylDPP-based polymers

**Scheme 2:**
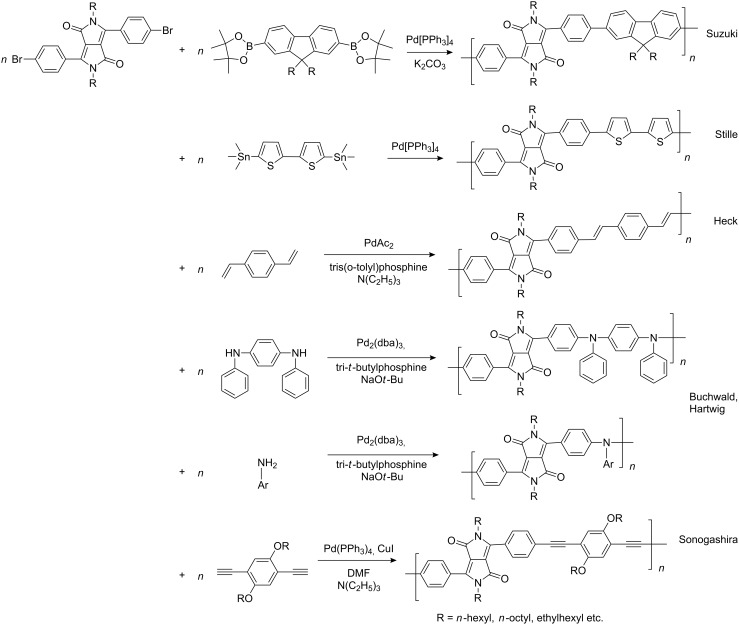
Pd-catalyzed coupling reactions for preparation of DPP-containing polymers.

In recent years a number of studies were reported on synthesis, optical, electrochemical, and electroluminescent properties of conjugated DPP polymers. The polymers were prepared by Suzuki, Heck, and Stille coupling and other catalytic polycondensation reactions. Typical examples are shown in Scheme 2. Rabindranath et al. [30] synthesized a new DPP polymer consisting entirely of aryl-aryl coupled diphenyl-DPP units (poly-DPP, **P-1**, see Table 1). The polymer was prepared by three different reactions. Pd-catalyzed and Ni-mediated one-pot coupling reactions were carried out starting from dibrominated DPP **M-1** as the sole monomer as well as conventional Pd-catalyzed coupling of **M-1** and the 3,6-diphenyl(4,4´-bis(pinacolato)boron ester) derivative of DPP. The polymer exhibits a bordeaux-red colour in solution with absorption maxima of about 525 nm, and a purple luminescence with a maximum around 630 nm with a Stokes-shift of about 105 nm. Cyclovoltammetric studies indicated quasi-reversible oxidation and reduction behaviour, the band gap being about 2 eV. Characteristic properties of **P-1** are listed in Table 1. In a comprehensive study, Zhu et al. prepared a number of highly luminescent DPP-based conjugated polymers [31]. The polymers consisted of dialkylated DPP units and carbazole, triphenylamine, benzo[2,1,3]thiadiazole, anthracene, or fluorene units in alternating fashion. They were prepared via Suzuki coupling, from the DPP monomers **M-1** or DPP-3,6-diphenyl(4,4´-bis(pinacolato)boron ester. A number of readily soluble polymers **P-2** to **P-8** exhibiting yellow to red absorption and emission colours, and fluorescence quantum yields of up to 86% were obtained. Characteristic properties are compiled in Table 1. Compared with the DPP monomers, the absorption of most of the polymers was bathochromically shifted by 24 to 39 nm. The small shift of **P-2** was ascribed to a large tilt angle between the π-planes of DPP and the adjacent comonomer units, in this case the anthracene unit, which strongly reduces the conjugation length [32]. EL devices prepared with **P-4** exhibited an external quantum efficiency (EQE) of 0.5% and a brightness at 20 V of 50 cd m^−2^ without much optimization. The maximum emission was at 600 nm, the turn-on voltage was 3.5 V. Cao et al. [33] prepared DPP-fluorene copolymers with a DPP content of between 0.1 and 50%. It was found that absorption and emission spectra, both in solution and thin film, varied regularly with the DPP content in the copolymers. On increasing the DPP content, the absorption only shifted by a few nanometers to longer wavelengths, whereas the emission was bathochromically shifted by more than 40 nanometers. EL properties of the copolymers were also studied: With increasing DPP content the EL colours varied from orange to red corresponding to CIE coordinates from (0.52, 0.46) to (0.62, 0.37). The best performance was achieved for an orange emitting device with a copolymer containing only 1% DPP units. The EQE was 0.45%, the maximum brightness 520 cd m^−2^. At high DPP content, the EQE was lowered to 0.14%, and the brightness to 127 cd m^−2^, similar to the results reported by Zhu et al. [31]. Cao et al. [34] also studied DPP-fluorene alternating copolymers with the fluorene unit being attached to the *m*-position of the phenyl groups in DPP (in contrast to the usual *p*-position). While the optical properties were quite similar, the EL properties were inferior. This was ascribed to a reduced conjugation length in these polymers.

**Table 1:** List of diphenylDPP-based polymers prepared upon Suzuki coupling and their characteristic properties.

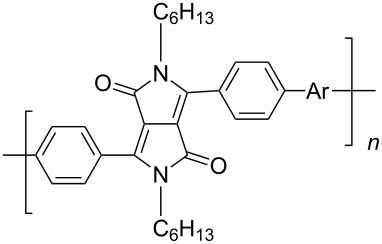								

Polymer	Ar	UV [nm] in solution	PL [nm] in solution	PL quantum yield Φ	HOMO [eV]	LUMO [eV]	MW [kDa]	Ref.

**P-1**	none	528	631	0.13	−5.39	−3.46	8.7	30
**P-2**	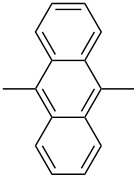	479	552	0.79	−5.60	−3.30	6.0	31
**P-3**	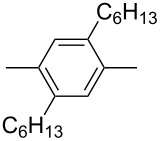	488	544	0.62	−5.40	−3.40	24.0	25
**P-4**	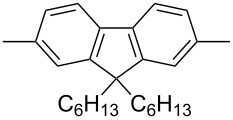	500	574	0.79	−5.30	−3.60	20.0	31
**P-5**	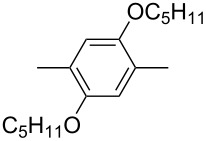	503	565	0.72	−5.30	−3.50	21.0	28
**P-6**	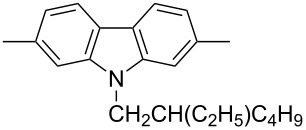	506	585	0.46	−5.33	−3.43	15.5	31
**P-7**	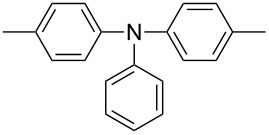	511	587	0.85	−5.37	−3.55	7.4	31
**P-8**	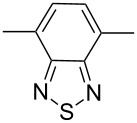	515	600	0.19	−5.57	−3.57	7.0	31

Novel vinyl ether-functionalized polyfluorenes for active incorporation in common photoresist materials were described by Kühne et al. [35] Among the polymers investigated was a diphenylDPP-fluorene copolymer, the fluorene units carrying ethyl vinylether groups in the 9,9´-position. The vinyl ether functionality allowed for active incorporation of the light emitting polymers into standard vinyl ether or glycidyl ether photoresist materials, the polymers retaining their solution fluorescence characteristics. This enabled photopatterning of light-emitting structures for application in UV-down-conversion, waveguiding, and laser media.

Using Stille coupling, Zhu et al. [36] first succeeded in the synthesis of copolymers **P-9** to **P-11** containing diphenylDPP and thiophene, bisthiophene, or 3,4-ethylenedioxythiophene (EDOT) units in alternating fashion (Table 2). Because of the strong donor-acceptor interaction between the thiophene and the DPP units, the absorption and emission maxima were shifted to longer wavelength: A solution of EDOT-DPP copolymer **P-11** exhibited a maximum absorption at 560 nm, and a solution-cast film of the same polymer had a λ_max_-value of 581 nm. The band gaps were between 1.5 and 1.7 eV, i.e., considerably smaller than for the previously reported DPP-based polymers. The fluorescence quantum yields Φ of the copolymers were rather weak (Φ ~15–35%), the maximum appeared at about 700–720 nm in the solid state. By Heck coupling it was possible to synthesize a polyarylenevinylene-type polymer **P-13**, the arylene units alternatingly being phenylenevinylene and diphenylDPP (Table 2) [36]. The polymer was obtained upon Pd-catalyzed reaction of dibromoDPP derivatives such as **M-1** and divinylbenzene. The resulting polymer had a molecular weight of about 30 kDa, was readily soluble in common organic solvents and its solutions exhibited a bright red colour with red light emission.

**Table 2:** List of DPP-polymers prepared upon Stille, Heck and Sonogashira coupling and their characteristic properties.

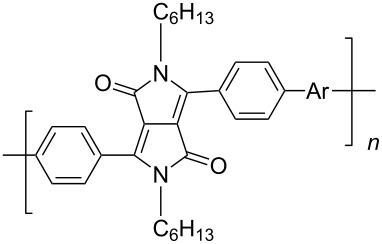								

Polymer	Ar	UV [nm] in solution	PL [nm] in solution	PL quantum yield Φ	HOMO [eV]	LUMO [eV]	MW [kDa]	Ref.

**P-9**	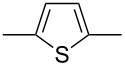	545/558	635/704	0.13	−5.26	−3.69	12.2	36
**P-10**	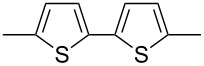	558/570	616/699	0.15	−5.15	−3.68	9.1	36
**P-11**	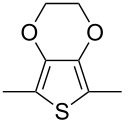	560/581	624/723	0.36	−5.411	−3.65	6.7	36
**P-12**	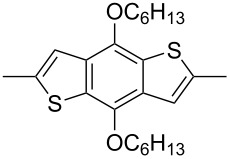	539/563	-	-	−5.47	−3.74	9.7	51
**P-13**	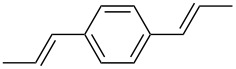	529/539	598/685	0.23	−5.75	−3.51	31.0	36
**P-14**	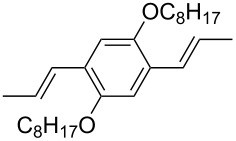	540/553	617/726	0.31	−5.61	−3.45	-	52
**P-15**	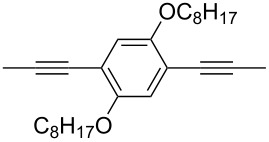	510/525	585/649	0.66	−5.80	−3.68	-	52

**Figure 3:**
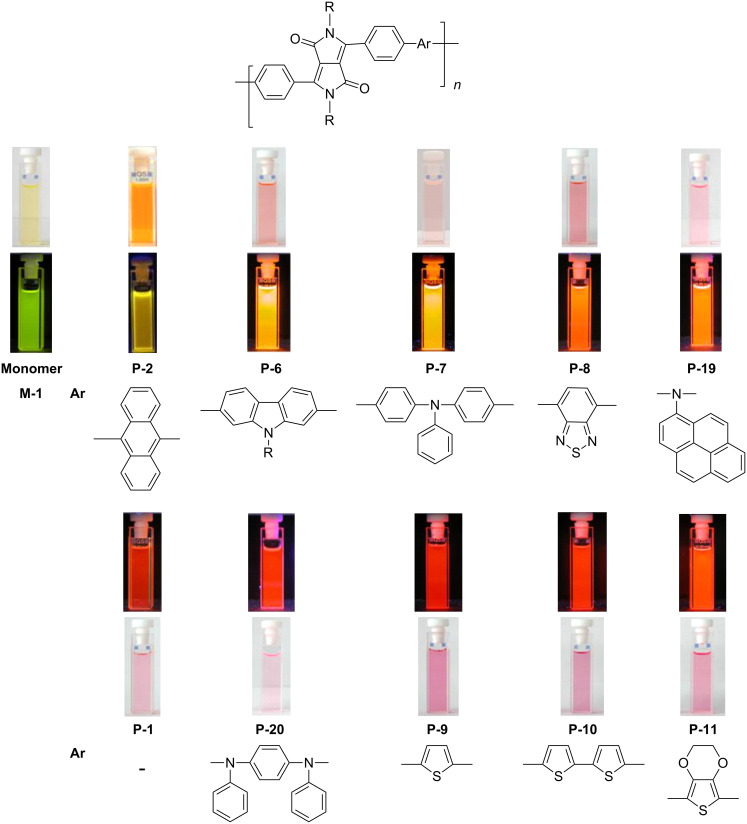
Optical properties of some diphenylDPP-based conjugated polymers.

A further study [37] focused on the incorporation of arylamine units in the main chain. Due to presence of electron-rich nitrogen atoms it was hoped that donor-acceptor interactions along the main chain would be enhanced and lead to a red-shift of the absorption and emission. Furthermore, the presence of easily oxidizable nitrogen in the main chain should give rise to a lower oxidation potential of the polymer. Relevant polymers **P-16** - **P-20** (Table 3) were synthesized using Pd-catalyzed aryl amination reactions as reported by Hartwig [38–39], Buchwald [40–42], and Kanbara [43–47]. As shown in Scheme 2, DPP monomers such as **M-1** were copolymerized with primary or secondary arylamines to yield DPP-containing polyiminoarylenes. The solutions of the polymers in chloroform exhibited a purple red colour with absorption maxima between 530 and 550 nm, and emission maxima from 610 to 630 nm. Fluorescence quantum yields were moderate (20 to 60%) (see also Table 3). The nitrogen atoms in the backbone lower the band gap of the polymers to approximately 1.9 eV. The band gaps are lower than for the conjugated DPP-arene copolymers prepared upon Suzuki coupling [31] but higher than for the DPP-thiophene copolymers made by Stille coupling [36]. Except for **P-16** and **P-18**, the polymers exhibit quasi reversible oxidation behaviour. A spectroelectrochemical study revealed that some of the polymers exhibited a reversible colour change between purple in the neutral state and a transparent greenish grey in the oxidized state. The electrochromism was very pronounced for **P-19** and **P-20**. Typical absorption and emission colours of several DPP-containing conjugated polymers are shown in Figure 3.

**Table 3:** List of DPP-based polyiminoarylenes and their characteristic properties [37].

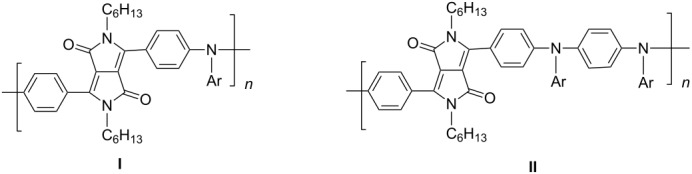								

Polymer	Ar	Type of polymer	UV [nm] in solution	PL [nm] in solution	PL quantum yield Φ	HOMO [eV]	LUMO [eV]	MW [kDa]

**P-16**	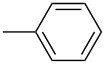	I	522/534	619/624	0.52	−5.24	−3.33	8.8
**P-17**	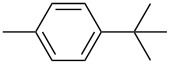	I	552/558	633/650	0.19	−5.41	−3.50	35.8
**P-18**	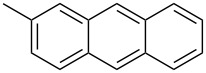	I	543/552	631/654	0.38	−5.41	−3.28	10.2
**P-19**	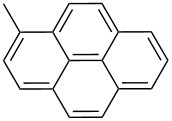	I	527/564	607	0.62	−5.24	−3.40	4.3
**P-20**	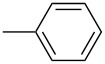	II	539/544	608	0.68	−5.06	−3.30	14.0

**Figure 4:**
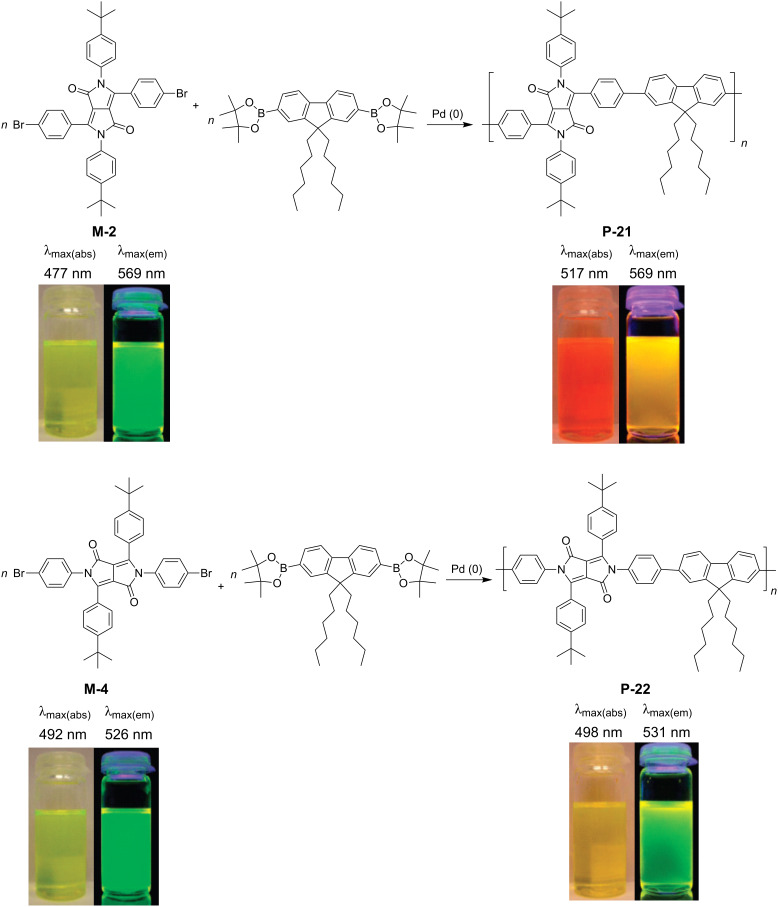
Optical properties of copolymers **P-21** and **P-22** based on two isomeric diphenylDPP monomer units (from [48]).

The synthesis of *N*-arylated diphenylDPP derivatives (also denoted as 2,3,5,6-tetraarylated DPP derivatives) such as **M-2** requires a different synthetic pathway outlined in Scheme 1. Direct *N*-arylation of the lactam group of DPP is only possible for activated arene units containing trifluoromethyl or nitro substituent groups. The common synthetic pathway first requires the synthesis of a diphenyldiketofurofuran derivative, which subsequently is reacted with an arylamine to yield the desired tetraarylated DPP derivative [11]. Using this approach, Zhang and Tieke [48] were able to prepare the two isomeric monomers **M-2** and **M-4** and their corresponding alternating copolymers **P-21** and **P-22** containing fluorene as the comonomer unit. While the properties of the two monomers are very similar, the optical and electrochemical properties of the two isomeric polymers are quite different. Suzuki coupling of **M-2** and a fluorene diboron ester derivative resulted in polymer **P-21** with fully conjugated main chain, the absorption being shifted by 15–25 nm compared with the monomer (Figure 4). The same coupling reaction of **M-4** resulted in polymer **P-22**, its π-conjugation being interrupted at the *N*-lactam units. Consequently, the absorption and emission behaviour were not much different from the corresponding monomer, the band gaps of the two isomers being 2 and 2.3 eV, respectively. The absorption and emission colours are shown in Figure 4.

**Figure 5:**
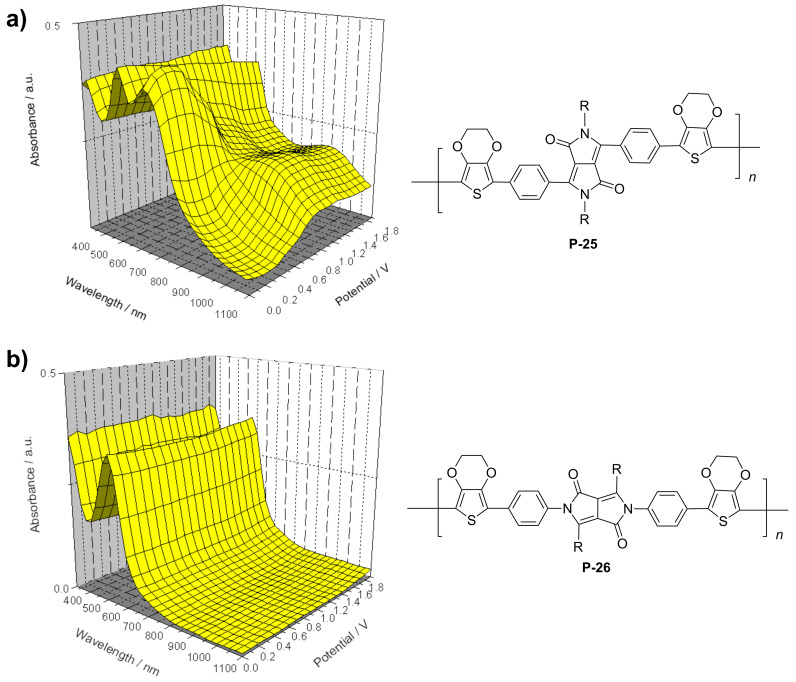
Absorption spectroelectrochemical plots of **P-25** and **P-26** as thin films on ITO glass. Scan rate: 100 mVs^−1^; potential vs. ferrocene (from [50]).

Stille coupling of **M-1** and 2-(tributylstannyl)-3,4-ethylenedioxythiophene gave the corresponding bis(thienyl)-substituted monomer [49]. Due to presence of the EDOT units, the monomer exhibited a rather low oxidation potential and could be easily electropolymerized by anodic oxidation. An insoluble, non-luminescent polymer film formed at the electrode that exhibited reversible electrochromic properties (Table 4). The film could be switched from blue in the neutral state via transparent grey to purple red in the oxidized state. The stability of the film was good, the switching could be repeated many times retaining 96% of the original absorption intensity after 100 cycles, without any protection against air or moisture. K. Zhang et al. [52] continued the studies and converted isomeric monomers **M-2** and **M-4** into corresponding bis-EDOT-substituted monomers. Both monomers could be electropolymerized, but the optical and electronic properties differed greatly between the two polymers. The polymers with EDOT-phenyl groups in the 3- and 6-positions (structure I in Table 4) represent conjugated polymers with low oxidation potentials and reversible electrochromic properties whereas the polymer with EDOT-phenyl groups in the 2- and 5- positions (structure II in Table 4) is non-conjugated, possesses a high oxidation potential and is not electrochromic (Figure 5).

**Table 4:** List of DPP-polymers prepared upon electrochemical polymerization.

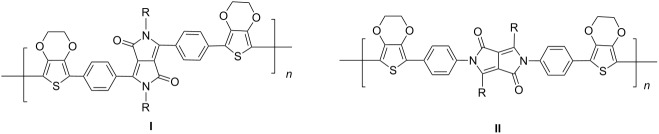										

Polymer	Type	R	λ_max_ of film [nm]	Half-wave oxidation potentials [V]				HOMO [eV]	LUMO [eV]	Ref.
				E_1_	E_2_	E_3_	E_4_			

**P-23**	I	*n*-hexyl	626	0.15	0.46	-	−1.70	−4.85	−3.39	49
**P-24**	I	(2-hexyl)decyl	588	0.28	0.54	0.97	−1.59	−4.88	−3.32	50
**P-25**	I	4-*t*-butylphenyl	648	0.21	0.50	-	−1.52	−4.85	−3.48	50
**P-26**	II	4-*t*-butylphenyl	510	1.61	-	-	-	−6.21	−2.89	50

Our activities have stimulated several other groups to synthesize diphenylDPP-containing conjugated polymers and to investigate their potential use in optoelectronic devices. Kanimozhi et al. [51] prepared alternating copolymers of diphenylDPP and 4,8-dihexylbenzo[1,2-b;3,4-b]dithiophene (**P-12**, Table 2) by Stille coupling and studied their optical and photovoltaic properties. Polymer-sensitized solar cells were fabricated with **P-12** as active layer. A power conversion efficiency of 1.43% was reached. G. Zhang et al. [52] synthesized diphenylDPP-containing polyphenylene-vinylene (PPV)- and polyphenylene-ethynylene (PPE)-type conjugated polymers via Heck- and Sonogashira coupling, respectively. PPV-type polymers such as **P-14** (Table 2) exhibit good solubility in common organic solvents, high thermal stability and a broad UV/visible absorption between 300 and 600 nm in films. Bulk heterojunction solar cells were fabricated and showed a power conversion efficiency of 0.01%. A PPE-type polymer such as **P-15** (Table 2) exhibited absorption and fluorescence maxima of 510 and 585 nm, respectively, the fluorescence quantum yield being 66%. Polymer/PCBM bulk heterojunction solar cells exhibited a power conversion efficiency of 0.16%. Cao et al. [53] prepared new fluorene-DPP-phenothiazine terpolymers by Suzuki coupling, and studied the EL properties. The best EL performance was achieved by a fluorene:DPP:phenothiazine 50:30.30 polymer with a maximum EQE of 0.25% and a maximum brightness of 259 cd m^−2^ in the device configuration of ITO/PEDOT/PVK/terpolymer/Ba/Al. DPP units effectively improved the electron affinity, and phenothiazine significantly enhanced the hole injection ability.

### ThiophenylDPP-based copolymers

**Scheme 3:**
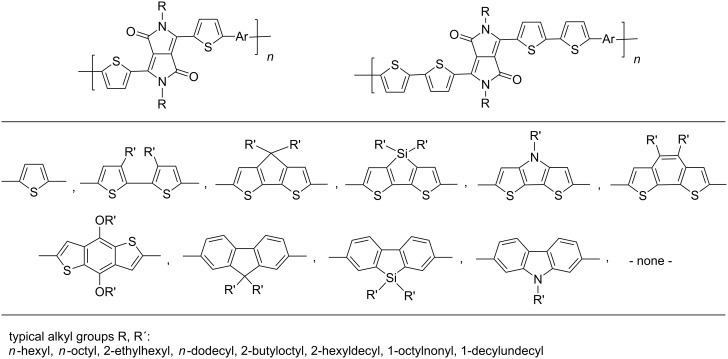
Thiophenyl-DPP-based polymers.

The replacement of the phenyl groups in 3,6-diphenyl-substituted DPP derivatives by thiophenyl groups resulted in 3,6-(2-thiophenyl)-substituted DPP derivatives (thiophenylDPPs) with absorption maxima at about 530 nm, i.e., more than 50 nm bathochromically shifted compared to diphenylDPP. Corresponding comonomer and polymer structures are listed in Scheme 3. Conjugated polymers containing thiophenylDPP in the main chain exhibited absorption maxima between 600 and 900 nm. Because of their small band gaps and high charge carrier mobilities, the polymers are interesting for applications in field effect transistors (FETs) and organic photovoltaic cells. Winnewisser et al. [54] succeeded in preparing the thiophenylDPP-based polymer, poly[3,6-bis(4´-dodecyl[2,2´]bithiophenyl)DPP] by Yamamoto coupling of a dibrominated thiophenylDPP derivative such as **M-3**. An ambipolar near-infrared light-emitting transistor (LET) could be prepared from this material which exhibited hole and electron mobilities of 0.1 cm^2^ V^−1^ s^−1^ and up to 0.09 cm^2^ V^−1^ s^−1^, respectively. These values were higher than any other ones previously reported for solution-processed ambipolar transistors. Janssen et al. [55] demonstrated the utility of thiophenylDPP-containing conjugated polymers for application in photovoltaic devices. From a mixture of C_70_PCBM and thiophenylDPP-based polymer **P-27** (Table 5) as active layer, solar cells with a power conversion efficiency up to 4.0% could be fabricated. The polymer exhibited a band gap of 1.4 eV, the maximum is shifted to 810 nm indicating chain aggregation and ordering, which is an important prerequisite for the preparation of films with good photovoltaic performance. In subsequent studies the efficiency could be further increased, e.g., by using **P-28** [56], or by the preparation of so-called ‘polymer tandem solar cells’ consisting of two subcells converting different parts of the solar spectrum [57]. For such a cell, an efficiency of 4.9% could be achieved (Table 6). Encouraged by the good performance of thiophenylDPP-based solar cells, further polymers **P-29** - **P-46** were recently synthesized and their photovoltaic properties investigated. Among these were alternating copolymers containing the thiophenylDPP, or bithiophenylDPP unit [58], and carbazole [58–59,61], fluorene [58,60–61,63], dibenzosilole, dithienosilole [58], benzo[1,2-b;3,4-b]dithiophene [63], benzo[2,1-b;3,4-b´]dithiophene [60], dithieno[3,2-b;2´,3´-d]pyrrole [61–62] and cyclopenta[2,1-b;3,4-b´]-dithiophene [63] as comonomer units (see Scheme 3). Some of the polymers were suited for the preparation of highly efficient polymer solar cells [60], some also exhibited ambipolar charge transport [62] with hole and electron transport mobilities up to 0.04 cm^2^ V^−1^ s^−1^ and 0.01 cm^2^ V^−1^ s^−1^, respectively [56]. Characteristic properties of photovoltaic devices are compiled in Table 6.

**Table 5:** Structure of thienyl-substituted DPP polymers used in photovoltaic devices.

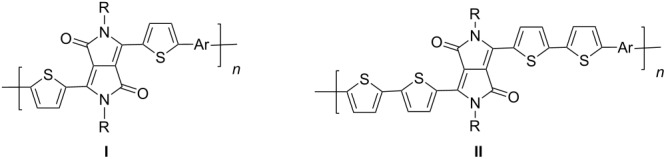					

Polymer	Type	R	Ar^a^	MW [kDa]	Ref.

**P-27**	I	2-ethylhexyl	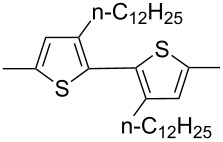	67	55
**P-28**	I	2-hexyldecyl	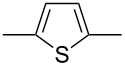	54	56
**P-29**	I	2-ethylhexyl	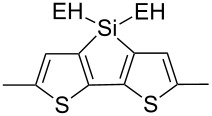	31.1	60
**P-30**	I	2-ethylhexyl	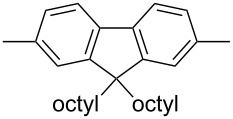	18.6	60
**P-31**	I	2-hexyldecyl	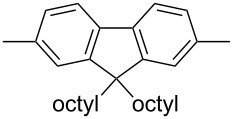	62	63
**P-32**	I	2-butyloctyl	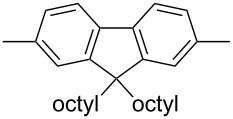	31	63
**P-33**	I	2-ethylhexyl	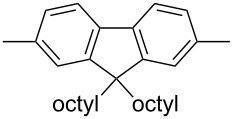	17	63
**P-34**	I	2-ethylhexyl	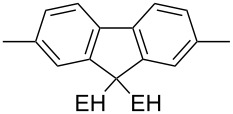	15.3	61
**P-35**	I	2-ethylhexyl	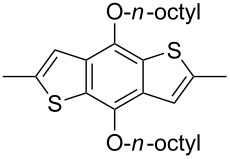	20.4	60
**P-36**	I	hexyl	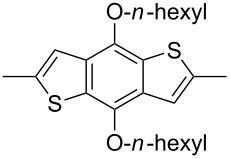	19	51,64
**P-37**	I	2-ethylhexyl	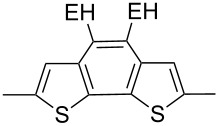	47.7	60
**P-38**	I	*n*-octyl	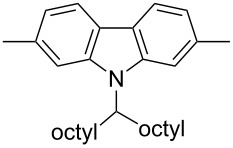	30	58,59
**P-39**	I	2-ethylhexyl	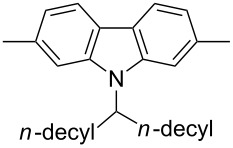	91.3	61
**P-40**	I	2-ethylhexyl	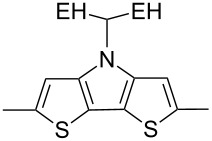	15.3	61
**P-41**	I	*n*-butyl	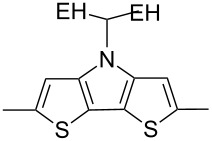	18.9	62
**P-42**	I	2-butyloctyl	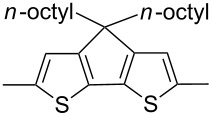	68	63
**P-43**	I	2-ethylhexyl	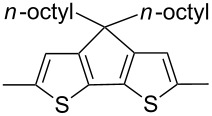	12	63
**P-44**	I	2-hexyldecyl	none	322	63
**P-45**	II	octyl	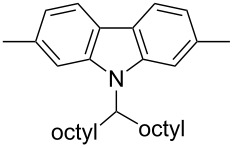	8	58
**P-46**	II	octyl	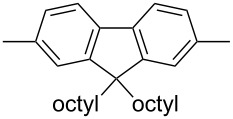	5	58

^a^EH = 2-ethylhexyl.

**Table 6:** Thienyl-substituted DPP polymers and their use in photovoltaic devices (properties that are of interest with regard to photovoltaic devices).

Polymer	λ_max_ (abs.) film [nm]	E_g_^el^ [eV]	HOMO(LUMO) [eV]	donor/PCBM ratio (w/w)	PCE [%]

**P-27**	-(810)	1.4	-	1:2	3.2
**P-28**	-	1.5	−5.17(−3.61)	1:2	4.9
**P-29**	798(796)	1.57	−5.04(−3.47)	1:2	2.1
**P-30**	649(652)	1.63	−5.23(−3.60)	1:2	0.78
**P-31**	663(664)	1.79	-	1:3	0.6
**P-32**	657(652)	1.79	-	1:4	0.8
**P-33**	658(656)	1.79	-	1:4	0.9
**P-34**	653(654)	1.78	−5.42(−3.64)	1:2	0.88
**P-35**	750(750)	1.65	−5.16(−3.51)	1:2	2.53
**P-36**	638(656)	1.46	−5.15(−3.69)	1:1	2.93
**P-37**	727(722)	1.58	−5.21(−3.63)	1:2	4.31
**P-38**	642(680)	1.52	−5.44(−3.92)	1:2	1.6
**P-39**	658(676)	1.69	−5.35(−3.62)	1:2	2.26
**P-40**	852(852)	1.17	−5.02(−3.64)	1:3	1.12
**P-41**	-(~770)	1.27	−4.90(−3.63)	1:2	2.71^a^
**P-42**	788(920)^b^	1.45	-	1:4	0.9
**P-43**	773(916)^b^	1.49	-	1:3	1.7
**P-44**	926(993)^b^	1.30	-	1:4	0.3
**P-45**	631(660)	1.46	−5.26(−3.80)	-	-
**P-46**	629(639, 678)	1.60	−5.40(−3.80)	-	-

^a^PC_70_BM; ^b^solution in o-dichlorobenzene, film cast from o-dichlorobenzene solution.

## Conclusion

Diaryldiketopyrrolopyrroles are insoluble red pigments, which on *N*-alkylation of the lactam groups and bromine substitution of the aryl groups can be converted into readily soluble monomers suitable for Pd-catalyzed polycondensation reactions. Using Suzuki, Stille, Heck and other aryl-aryl coupling reactions, new conjugated polymers with good solubility in common organic solvents, high molecular weight, high thermal stability and application potential for optoelectronic devices became accessible. Polymers containing diphenylDPP units in the main chain exhibit brilliant orange, red or purple colours, intense luminescence, high luminescence quantum yields, and Stokes-shifts up to 110 nm. Some of the polymers were studied as active layers in electroluminescent devices and showed a brightness up to 500 cd m^−2^. Polymers with dithiophenylDPP moieties in the main chain show broad absorption in the visible exhibiting blue or dark green colours, small band gaps and high charge carrier mobilities. They are suitable as electron donor in bulk heterojunction solar cells with PC_60_BM or PC_70_BM as electron acceptors to give maximum power conversion efficiencies of about 5%. In field-effect transistors they exhibit ambipolar charge transport with large hole and electron mobilities. Variation of comonomer units or aryl groups in DPP monomers might further improve the device properties.
